# Reliability and validity of a Korean version of the children’s eating behavior questionnaire in anorexia context

**DOI:** 10.3389/fnut.2023.1247630

**Published:** 2023-11-01

**Authors:** Mi Mi Ko, Sun Haeng Lee, Gyu Tae Chang, Boram Lee

**Affiliations:** ^1^KM Science Research Division, Korea Institute of Oriental Medicine, Daejeon, Republic of Korea; ^2^Department of Korean Pediatrics, College of Korean Medicine, Kyung Hee University Medical Center, Kyung Hee University, Seoul, Republic of Korea; ^3^Department of Korean Pediatrics, College of Korean Medicine, Kyung Hee University Hospital at Gangdong, Kyung Hee University, Seoul, Republic of Korea

**Keywords:** Childhood anorexia, eating behavior, Children’s eating behavior questionnaire, anorexia, CEBQ

## Abstract

**Background:**

Although childhood anorexia is a common clinical disorder, there is no established tool for evaluating it. The Children’s Eating Behavior Questionnaire (CEBQ) is a parent-reported measure designed to assess the eating behavior of children. We aimed to investigate the reliability and validity of the Korean version of the CEBQ (K-CEBQ) for children with anorexia.

**Methods:**

Parents of children with anorexia aged between 2 and 9 years participated in a survey conducted twice at 1-month intervals. The general characteristics and K-CEBQ scores of the children were recorded. To assess the reliability of the K-CEBQ, the internal consistency and test–retest methods were used. Furthermore, correlation analysis was performed for each item and factor, and the discriminant validity was determined through comparison with the normal group. Optimal cut-off scores, which are based on the maximum area under the curve of the receiver operating characteristic curve, were calculated in two categories.

**Results:**

A total of 336 participants responded to the first survey, and the responses from 171 participants from the second survey were included in the analysis. The K-CEBQ showed relatively high internal consistency reliability (Cronbach’s alpha = 0.738), and the retest demonstrated sufficient temporal stability. The relationship between each item of the K-CEBQ and the factor to which the item belongs showed a high correlation. There were significant differences between the anorexia and normal groups in two categories of the K-CEBQ: ‘food approach’ (*p* = 0.0063) and ‘food avoidant’ (*p* < 0.0001). The optimal cut-off values for the ‘food approach’ and ‘food avoidant’ category scores were 39.50 and 58.50 points, respectively.

**Conclusion:**

This study demonstrated that the K-CEBQ may be a reliable and valid tool for assessing the eating behavior of children with anorexia.

## 1. Introduction

Childhood anorexia is a common disorder characterized by a long-term decrease in food intake, a lack of interest in food, and a pattern of food refusal in severe cases, and anorexia in children is mostly classified as idiopathic (1). The prevalence of childhood anorexia is estimated to be 14–50% among preschool-aged children and 7–27% among school-aged children, with a reported increase in recent years (2). Although the overall prognosis is usually favorable, prolonged anorexia can cause malnutrition, hinder children’s growth and development, and weaken their immune system, making them susceptible to various illnesses (3, 4). However, as anorexia in children without any identifiable organic cause is primarily based on subjective symptoms or signs, it is challenging to determine the severity of anorexia or establish treatment strategies in research or clinical settings. To address this issue, several tools have been developed to measure the eating behavior of children (5–9). Among them, the Children’s Eating Behavior Questionnaire (CEBQ) is generally considered as one of the easiest questionnaires for measuring children’s eating behavior. The CEBQ is a multifaceted 35-item questionnaire for parents to measure individual differences in eating behavior that are thought to result in underweight and overweight children (9–13). In South Korea, the questionnaire has been officially translated into a Korean version, and the reliability and validity were confirmed for children with normal development (11). The total CEBQ score of anorexic children has been reported to be lower than that of non-anorexic children (14). However, the reliability and validity of the CEBQ for children with anorexia have not been thoroughly investigated. Furthermore, the previous study (14) did not provide information on cut-off values for diagnosing anorexia or determining the treatment progress. Therefore, we aimed to investigate the reliability and validity of the Korean version of the CEBQ (K-CEBQ) for children with anorexia. In addition, we calculated cut-off scores in two categories of the K-CEBQ to aid clinical decision-making for diagnosing children with anorexia and assessing their treatment progress.

## 2. Methods

### 2.1. Study design

This study was based on a survey of parents of children with anorexia. The survey was conducted by EMBRAIN PUBLIC^1^, a company specializing in public surveys. The company sent a survey via SMS or e-mail to a list of registered members, which contained a detailed description of the research including its purpose, contents, and potential benefits and harms. Participants who voluntarily agreed to participate in the study were recruited on a first-come, first-served basis. The survey was anonymously conducted twice for the same participants at intervals of 1 month between April 1 and May 13, 2022.

### 2.2. Participants

**Figure 1:**
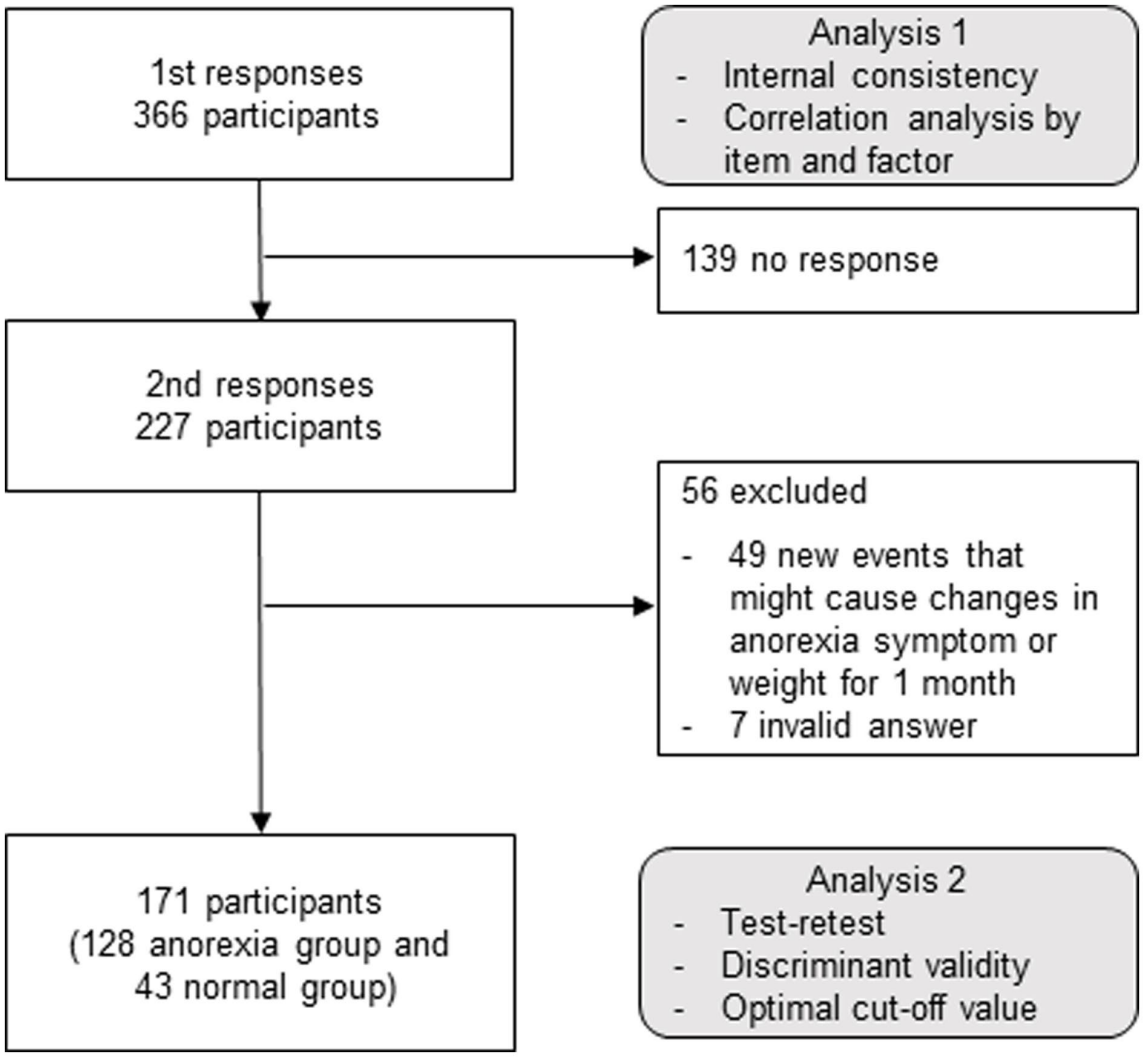
Study flow chart. K-CEBQ, Korean version of the children’s eating behavior questionnaire.

Parents of children with anorexia were included in the survey based on the inclusion criteria of Diagnostic Classification of Mental Health and Developmental Disorders of Infancy and Early Childhood (DC:0–3R) (16) as follows: (1) Refusal to eat an adequate amount of food for more than 1 month; (2) Lack of hunger and interest in food; (3) Food refusal is not caused by trauma or an underlying medical condition; (4) Between the ages of 2 and 9 years. If the respondent had two or more children with anorexia, the response was based on the child with the most severe symptoms. An initial sample was selected to reach the desired sample size of 250 participants with survey completion. This sample size provides a margin of error for estimated rates of ±6.2 percentage points, conservatively assuming a population rate of 50% and a 95% confidence level (17). For the first survey, 336 participants responded without interruptions or omissions. One month later, a second questionnaire was sent to the same respondents. Participants with children who had no new events that might cause changes in anorexia symptoms or weight for 1 month, did not receive any new treatment for anorexia, and had no missing data were included in the analysis. If the symptoms of anorexia disappeared in the second survey, they were classified as the normal group. A total of 227 participants responded to the second survey, and 56 of them were excluded because of new events that might cause changes in anorexia symptoms or weight for 1 month, including enteritis, influenza, transfer to another school, and coronavirus disease 2019, or invalid answers. As a result, the responses from 171 participants (128 participants in the anorexia group and 43 participants in the normal group) were analyzed (Figure 1).

### 2.3. Measures: 1st survey

The first survey included the following measures for children with anorexia:

(1) General characteristics including age, sex, weight, height, and history of anorexia treatment in the past 1 month were collected. In the case of weight, to ensure the accuracy of the data, the respondents were asked to upload a photo showing their weight along with the date of measurement after measuring the weight using a scale.

(2) K-CEBQ: this is a parent-reported questionnaire to assess children’s eating behavior. It consists of 35 items and measures the ‘food approach’ and ‘food avoidant’ behavior categories. In the ‘food approach’ behavior category, a total of 4 factors are included: food responsiveness (FR), enjoyment of food (EF), emotional overeating (EOE), and desire to drink (DD). In the ‘food avoidant’ behavior category, a total of 4 factors are included: satiety responsiveness (SR), slowness in eating (SE), emotional undereating (EUE), and food fussiness (FF). Each question is scored on a 5-point Likert-type scale (1 = never, 2 = rarely, 3 = sometimes, 4 = often, 5 = always). Scores corresponding to each category and individual factors were calculated by adding the sum of the scores for each item. Currently, the CEBQ has been officially translated into Korean (11). Our research team obtained permission to use the questionnaire for academic purposes from the original developer of the instrument (9) and from researchers who officially translated it into Korean (11).

### 2.4. Measures: 2nd survey

The second survey, conducted 1 month later, included the following measures for children with anorexia:

1. Persistence of anorexia symptoms for 1 month.
2. Occurrence of new events that may cause a change in anorexia symptoms and weight for 1 month.
3. History of anorexia treatment in the past month.
4. K-CEBQ.

### 2.5. Statistical analysis

All statistical analyses were conducted using a two-tailed approach, and the significance level was set at 0.05. To assess the reliability of the K-CEBQ, we calculated the internal consistency coefficient (Cronbach’s alpha) and employed the test–retest method to ensure the stability of questionnaire scores over time. The mean value and standard deviation (SD) were calculated for each factor of the K-CEBQ. An independent sample t-test was conducted to investigate whether there were differences in the factor scores based on demographic variables such as sex and age following a Kolmogorov–Smirnov test to confirm the normality of the data distribution. To determine whether each item accurately predicts the score of the corresponding factor, Pearson’s correlation analysis was conducted for each item, and the correlation coefficients between factors were obtained. In addition, to verify the discriminant validity of the test, differences between anorexic children and normal children were examined by independent t-test. The optimal cut-off scores for the ‘food avoidant’ and ‘food approach’ behavior, which are based on the maximum area under the curve (AUC) of the receiver operating characteristic (ROC) curve obtained by calculating the sensitivity and specificity of a test, were determined. To minimize potential confounding factors, propensity score matching (PSM) was applied using a 1:1 nearest-neighbor matching algorithm to adjust for age and sex.

### 2.6. Ethical consideration

This study received an exemption for review from the Institutional Review Board of the Korea Institute of Oriental Medicine (No. I-2109/008–002, October 13, 2021) as the study did not collect any identifiable information from the participants.

## 3. Results

### 3.1. General characteristics

Among the 366 participants who responded to the first survey, 267 (72.95%) and 99 (27.05%) of them were mothers and fathers, respectively. Based on the data on the sex of their children, there were 213 boys (58.2%) and 153 girls (41.8%). The average age of the children was 5.57 ± 1.97 years. The average weight and height of the children were 18.89 ± 4.90 kg and 109.0 ± 13.81 cm, respectively. The average weight-for-age percentile was 38.31 ± 26.77 for boys and 40.10 ± 28.55 for girls. The average weight-for-height percentile was 71.82 ± 35.21 for boys and 73.11 ± 35.71 for girls.

A total of 32 children (8.74%) had been treated for anorexia within the past month. Among them, the most commonly used treatment was nutritional supplements (*n* = 21), followed by herbal medicine (*n* = 18) and conventional medication (*n* = 8) (Table 1).

**Table 1:** General characteristics.

		Anorexia children		
		Total (*N* = 366)	Boys (*N* = 213)	Girls (*N* = 153)
Respondent	Father/mother (of children)	99/267	57/156	42/111
Children	Age (years)	5.57 ± 1.97	5.55 ± 1.98	5.60 ± 1.96
	Weight (kg)	18.89 ± 4.90	18.95 ± 4.91	18.81 ± 4.89
	Height (cm)	109.0 ± 13.81	109.20 ± 13.61	108.70 ± 14.11
	Weight-for-age (percentile)	39.06 ± 27.50	38.31 ± 26.77	40.10 ± 28.55
	Weight-for-height (percentile)	72.36 ± 35.35	71.82 ± 35.21	73.11 ± 35.71
History of anorexia treatment	Yes (%)	32 (8.74)	19 (8.92)	13 (8.50)
Type of treatment^1^	Nutritional supplements	21 (65.63)	10 (52.63)	11 (84.62)
	Conventional medication	8 (25.0)	5 (26.32)	3 (23.08)
	Herbal medicine	18 (56.25)	10 (52.63)	8 (61.54)
	Others^2^	4 (12.50)	2 (10.53)	2 (15.38)

All data are presented as mean ± SD for continuous variables and as frequencies (%) for categorical variables.

^1^Multipe responses allowed.

^2^Acupuncture, moxibustion, cupping therapy, etc.

### 3.2. Reliability

To evaluate the internal consistency of the K-CEBQ for each factor, reliability analysis was conducted based on 366 responses, and the reliability coefficient (Cronbach’s alpha) of the total items was 0.738. In addition, to examine the temporal stability of the questionnaire, a retest was conducted on 128 respondents having children with anorexia with an interval of 26 to 37 days from the first survey (mean 30.20, SD 2.71). The correlation coefficient of the total items was 0.712, indicating an appropriate level of reliability as shown in Table 2.

**Table 2:** Reliability of K-CEBQ (*N* = 366).

K-CEBQ	Items	Cronbach’s alpha	Test-retest^1^
**‘Food approach’**	17	0.732	0.769
Food responsiveness (FR)	5	0.731	0.740
Enjoyment of food (EF)	5	0.741	0.660
Emotional overeating (EOE)	4	0.734	0.716
Desire to drink (DD)	3	0.716	0.776
‘**Food avoidant’**	18	0.710	0.690
Satiety responsiveness (SR)	5	0.715	0.652
Slowness in eating (SE)	4	0.731	0.730
Emotional undereating (EUE)	4	0.709	0.698
Food fussiness (FF)	5	0.746	0.611

^1^*N* = 128, All value of *p* < 0.0001. K-CEBQ, the Korean version of children’s eating behavior questionnaire.

### 3.3. Correlation analysis according to item and factor

To investigate the relationship between each item of the K-CEBQ and its corresponding factor, correlation analysis was performed. The results showed that all item correlations were statistically significant (*p* < 0.001), and the correlation coefficients were high (*r* = 0.516–0.911) (Table 3). In addition, to determine the correlation between the factors of the K-CEBQ, the factors were divided into two categories (‘food approach’ behavior and ‘food avoidant’ behavior) according to the measurement content, and the correlation between the factors in each category was analyzed. Overall, Pearson’s correlation coefficients between factors showed statistically significant positive correlations within categories measuring the same content. On the other hand, there were mostly negative correlations between the factors of the ‘food approach’ and ‘food avoidant’ categories (Table 4).

**Table 3:** Correlation analysis by item and factor of K-CEBQ (*N* = 366).

Item		*r*
**‘Food approach’**	Food responsiveness (FR)	
	My child is always asking for food	0.676*
	If allowed to, my child would eat too much	0.668*
	If given the chance, my child would always have food in his/her mouth	0.715*
	Even if my child is full up, s/he finds room to eat his/her favorite food	0.639*
	Given the choice, my child would eat most of the time	0.704*
	Enjoyment of food (EF)	
	My child loves food	0.728*
	My child has a big appetite	0.734*
	My child is interested in food	0.753*
	My child looks forward to mealtimes	0.748*
	My child enjoys eating	0.820*
	Emotional overeating (EOE)	
	My child eats more when worried	0.715*
	My child eats more when annoyed	0.808*
	My child eats more when anxious	0.824*
	My child eats more when s/he has nothing else to do	0.756*
	Desire to drink (DD)	
	My child is always asking for a drink	0.804*
	If given the chance, my child would drink continuously throughout the day	0.911*
	If given the chance, my child would always be having a drink	0.908*
**‘Food avoidant’**	Satiety responsiveness (SR)	
	My child leaves food on his/her plate at the end of a meal	0.682*
	My child gets full before his/her meal is finished	0.729*
	My child is difficult to please with meals	0.516*
	My child gets full up easily	0.752*
	My child cannot eat a meal if s/he has had a snack just before	0.686*
	Slowness in eating (SE)	
	My child finishes his/her meal very quickly (R)	0.714*
	My child eats slowly	0.827*
	My child takes more than 30 min to finish a meal	0.828*
	My child eats more and more slowly during the course of a meal	0.775*
	Emotional undereating (EUE)	
	My child eats less when s/he is angry	0.800*
	My child eats less when s/he is tired	0.738*
	My child eats more when s/he is happy	0.593*
	My child eats less when s/he is upset	0.825*
	Food fussiness (FF)	
	My child refuses new foods at first	0.753*
	My child enjoys tasting new foods (R)	0.802*
	My child enjoys a wide variety of foods (R)	0.745*
	My child is interested in tasting food s/he has not tasted before (R)	0.747*
	My child decides that s/he does not like food, even without tasting it	0.660*

(R), reverse scored item; *p < 0.001 (two-sided); r, Pearson’s correlation value.

**Table 4:** Correlations between the K-CEBQ factors (*N* = 366).

K-CEBQ									
		FR	EF	EOE	DD	SR	SE	EUE	FF
**‘Food approach’**	Food responsiveness (FR)	-							
	Enjoyment of food (EF)	0.65***	**-**						
	Emotional overeating (EOE)	0.64***	0.52***	**-**					
	Desire to drink (DD)	0.22***	0.14**	0.14**	-				
‘**Food avoidant’**	Satiety responsiveness (SR)	−0.19***	−0.29***	−0.09*	0.17***	-			
	Slowness in eating (SE)	−0.20***	−0.32***	−0.29***	0.07	0.39***	**-**		
	Emotional undereating (EUE)	0.06	0.17**	0.09	0.17**	0.32***	0.14**	**-**	
	Food fussiness (FF)	−0.37***	−0.56***	−0.29***	0.02	0.32***	0.20***	**0.06**	-

*p < 0.05; **p < 0.01; ***p < 0.001 (two-sided); Bold values indicate Pearson’s correlations between factors belonging to the same category. K-CEBQ, the Korean version of children’s eating behavior questionnaire.

### 3.4. Sex and age differences of K-CEBQ

There were no significant differences between boys and girls in all factors of the K-CEBQ except for the SE factor. Additionally, to investigate whether there were any differences in K-CEBQ scores based on age, the sample was divided into two groups (under 6 years and 6 years or older). The results showed that there were significant differences in scores of the ‘food avoidant’ category, as well as the DD and EUE factors (Supplementary Tables S1, S2).

### 3.5. Discriminant validity

The discriminant validity of the K-CEBQ was assessed by t-test between a group with persistent anorexia symptoms and a group with no anorexia symptoms in the second survey after 1 month. The results showed significant differences between the anorexia group and the normal group in the two categories, i.e., ‘food approach’ (*p* = 0.0063) and ‘food avoidant’ (*p* < 0.0001), of the K-CEBQ. Significant differences were observed between the two groups in all factors except for EOE and DD in the ‘food approach’ category and EUE in the ‘food avoidant’ category (Table 5). Analysis of differences between the anorexia and normal groups after PSM according to sex and age (n = 43 per group) also revealed significant differences between the groups in the ‘food approach’ (*p* = 0.0021) and ‘food avoidant’ (*p* = 0.0002) categories. Following PSM, significant differences were observed between the two groups in all factors except for DD in the ‘food approach’ category and EUE in the ‘food avoidant’ category (Supplementary Table S3).

**Table 5:** Comparison of the K-CEBQ between anorexia and normal groups.

Variables	Total (*N* = 171)	Anorexia (*N* = 128)	Normal (*N* = 43)	value of *p*
Sex (M/F)	91/80	70/58	21/22	0.5059
Age (years)	5.75 ± 1.86	5.65 ± 1.90	6.07 ± 1.71	0.1987
**K-CEBQ**				
**‘Food approach’**	30.95 ± 7.92	29.77 ± 6.71	34.44 ± 10.06	**0.0063**
Food responsiveness (FR)	7.76 ± 2.64	7.30 ± 2.22	9.14 ± 3.27	**0.0011**
Enjoyment of food (EF)	9.77 ± 3.04	11.63 ± 3.45	11.63 ± 3.45	**<0.0001**
Emotional overeating (EOE)	5.61 ± 2.17	5.41 ± 1.94	6.23 ± 2.68	0.0674
Desire to drink (DD)	7.8 ± 3.19	7.92 ± 3.3	7.44 ± 2.81	0.3948
**‘Food avoidant’**	63.56 ± 9.17	65.89 ± 8.16	56.6 ± 8.56	**<0.0001**
Satiety responsiveness (SR)	16.78 ± 3.55	17.72 ± 3.28	14.0 ± 2.81	**<0.0001**
Slowness in eating (SE)	15.93 ± 3.12	16.48 ± 3.03	14.3 ± 2.84	**<0.0001**
Emotional undereating (EUE)	11.67 ± 3.84	11.86 ± 4.03	11.12 ± 3.16	0.2730
Food fussiness (FF)	19.17 ± 3.69	19.84 ± 3.39	17.19 ± 3.88	**<0.0001**

value of *p* by independent t-test. K-CEBQ, the Korean version of children’s eating behavior questionnaire.

### 3.6. Optimal cut-off value

**Figure 2:**
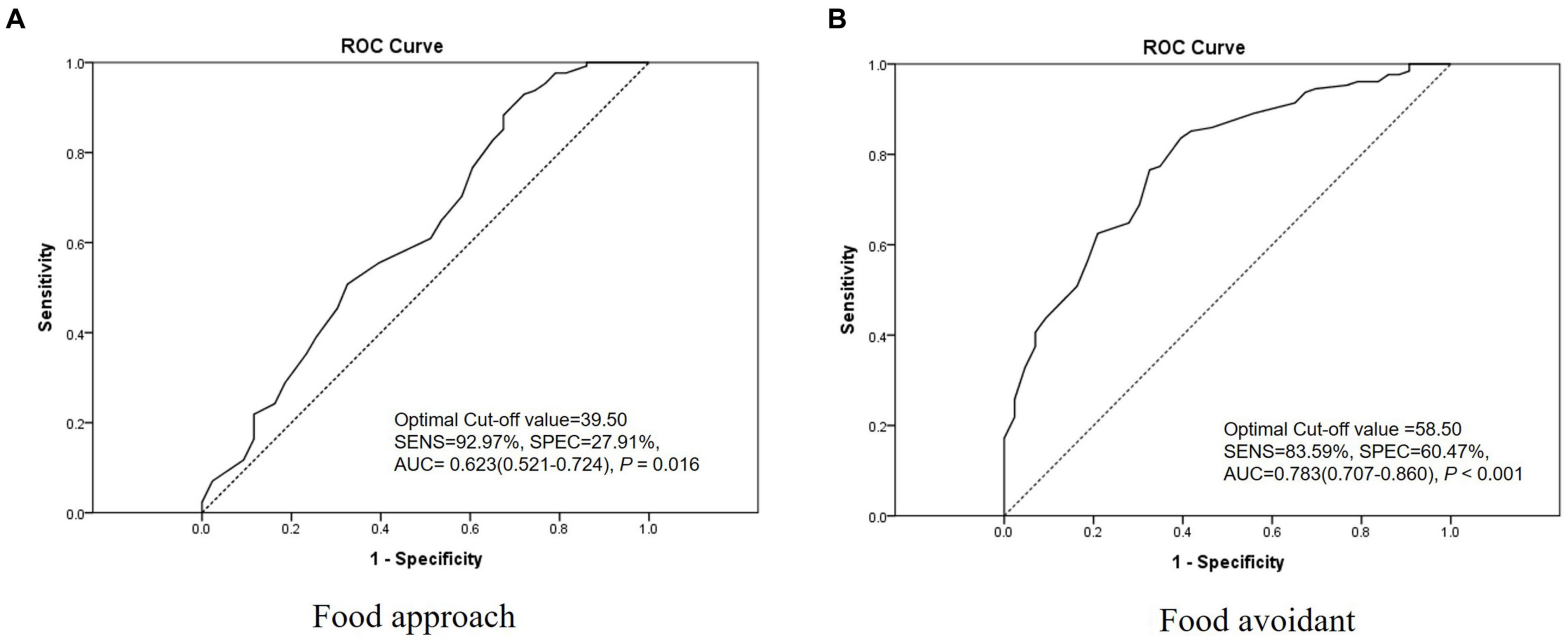
Receiver operating characteristic curve of the K-CEBQ. AUC, area under the curve; K-CEBQ, Korean version of the children’s eating behavior questionnaire; ROC, receiver operating characteristic; SENS, sensitivity; SPEC, specificity.

**Figure 3:**
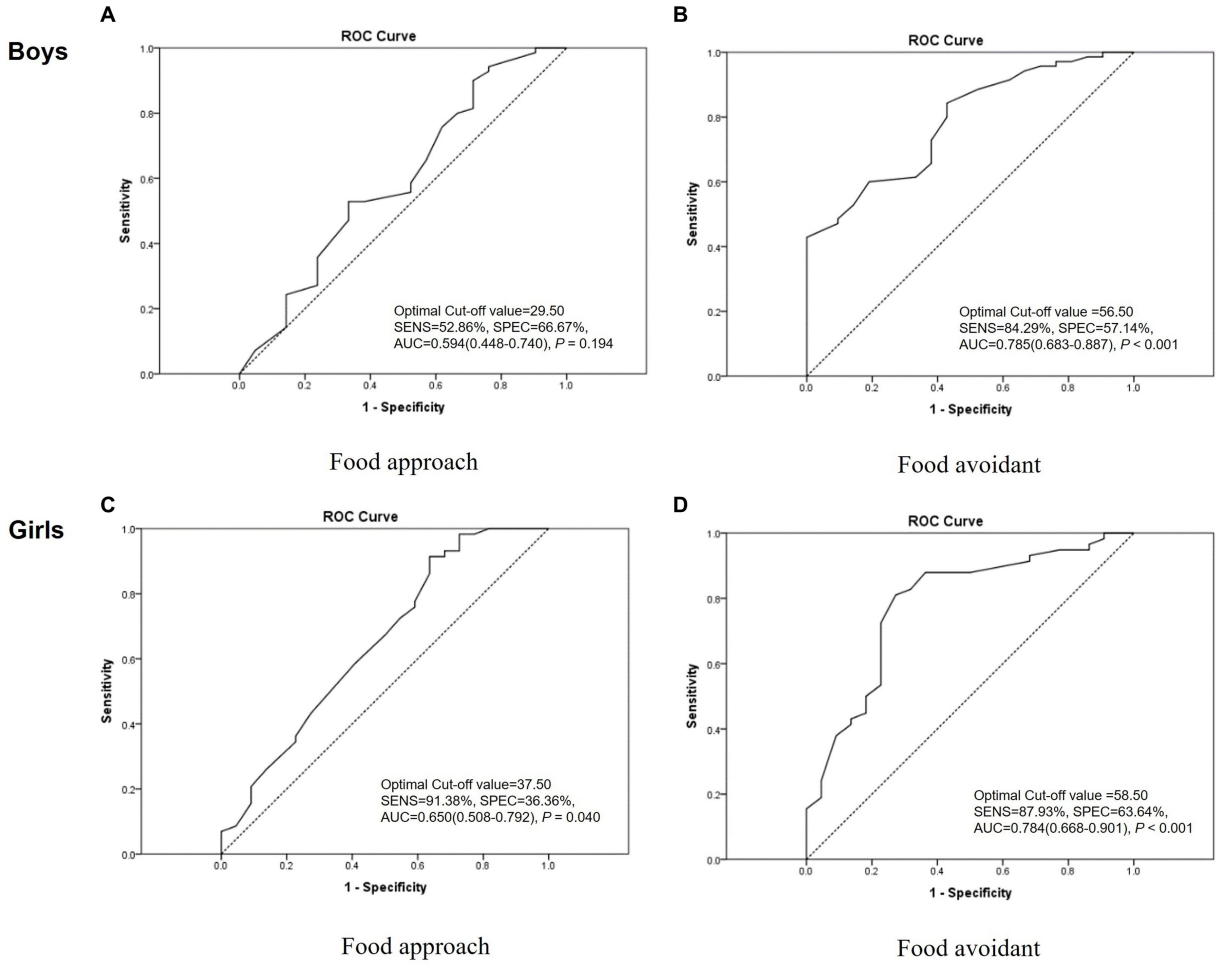
Receiver operating characteristic curve of the K-CEBQ according to sex. AUC, area under the curve; K-CEBQ, Korean version of the children’s eating behavior questionnaire; ROC, receiver operating characteristic; SENS, sensitivity; SPEC, specificity.

**Figure 4:**
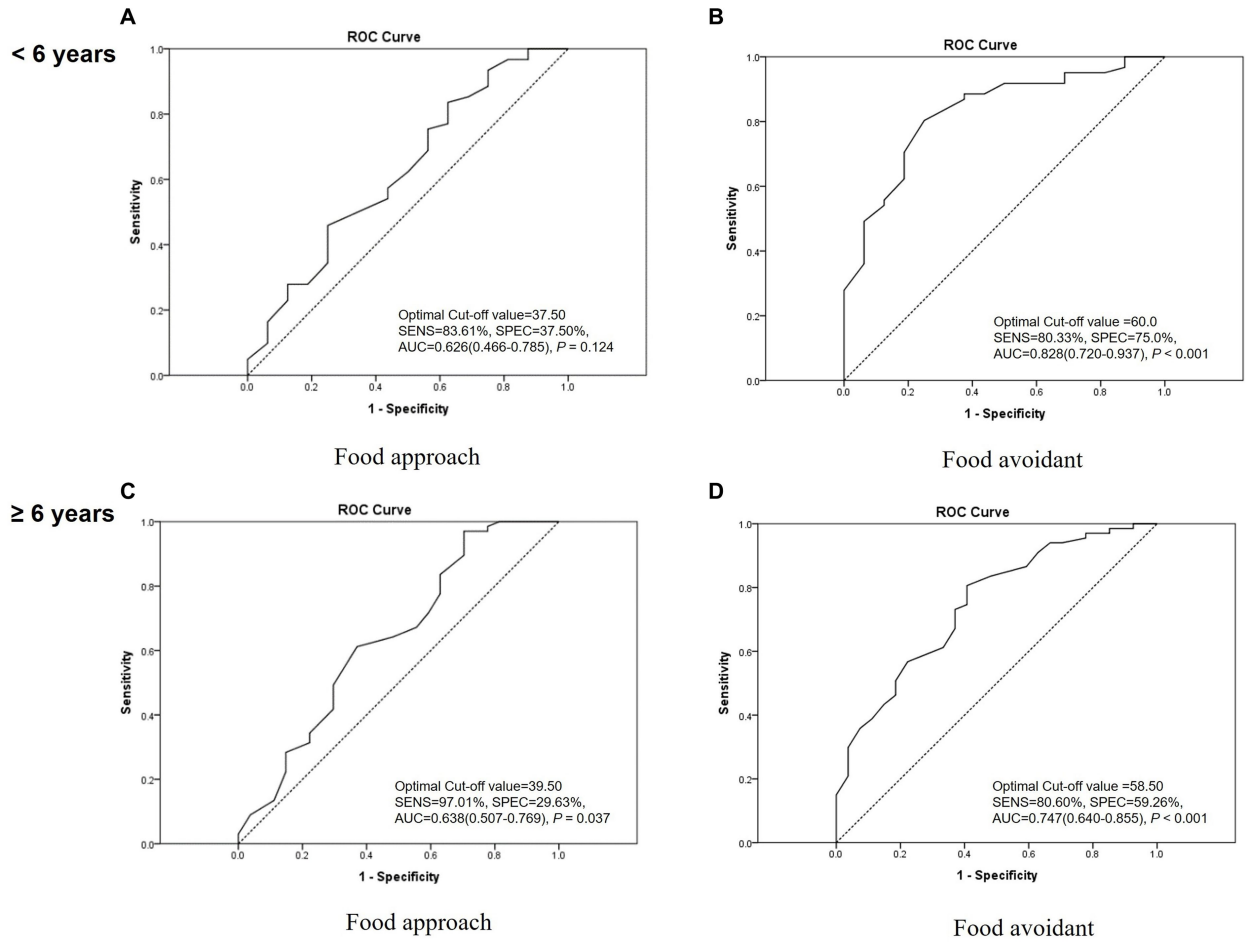
Receiver operating characteristic curve of the K-CEBQ according to age. AUC, area under the curve; K-CEBQ, Korean version of the children’s eating behavior questionnaire; ROC, receiver operating characteristic; SENS, sensitivity; SPEC, specificity.

The optimal cut-off values for the ‘food approach’ and ‘food avoidant’ category scores were 39.50 and 58.50 points, respectively. Evaluation of diagnostic accuracy using the cut-off values showed a sensitivity of 92.97% and a specificity of 27.91% for the ‘food approach’ category and a specificity of 83.59% and a specificity of 60.47% for the ‘food avoidant’ category [AUC (95% CI, value of *p*); 0.623 (0.521–0.724, *p* = 0.016) and 0.783 (0.707–0.860, *p* < 0.001), respectively] (Figure 2). The optimal cut-off scores for the ‘food approach’ and ‘food avoidant’ categories showed slightly different results according to sex and age (Figures 3, 4). After PSM according to sex and age, the optimal cut-off values for the ‘food approach’ and ‘food avoidant’ category scores were 29.50 and 58.50 points, respectively. Diagnostic accuracy measurements demonstrated 62.79% sensitivity and 67.44% specificity for the ‘food approach’ category [AUC (95% CI, value of *p*); 0.674 (0.561–0.788, *p* < 0.005)] and 76.74% sensitivity and 60.47% specificity for the ‘food avoidant’ category [AUC (95% CI, value of *p*); 0.720 (0.612–0.827, *p* < 0.001)] (Supplementary Figure S1). The optimal cut-off scores for the ‘food approach’ and ‘food avoidant’ categories also showed different results according to sex and age after PSM (Supplementary Figures S2, S3).

## 4. Discussion

Anorexia is a common disorder with high prevalence among children (2). The diagnostic method frequently used for children with anorexia is based on clinical features, and the diagnostic tools frequently used are height and weight measurement and bioelectrical impedance analysis (18). The K-CEBQ is a representative tool for evaluating the eating behavior of children and has been actively used in clinical settings (9). However, to the best of our knowledge, studies on its reliability, validity, and cut-off scores for children with anorexia have not been thoroughly conducted. The present study aimed to evaluate the reliability and validity of the K-CEBQ for children with anorexia and to calculate cut-off scores to aid clinical decision-making.

According to our results, the K-CEBQ exhibited good internal consistency reliability and temporal stability, indicating its reliability for assessing the eating behavior of children with anorexia. Specifically, the calculated Cronbach’s alpha coefficient was 0.738; as a Cronbach’s alpha value of 0.60 or higher indicates internal consistency (19), the questionnaire showed high reliability.

The relationship between each item of the K-CEBQ and the factor to which the item belongs also showed a high correlation, and factors within each category were significantly correlated, suggesting that the questionnaire could measure distinct dimensions of eating behavior for children with anorexia. As there are cultural differences in intake-related behavior (20), we followed the 8-factor structure, which is established following factor analysis of the officially translated K-CEBQ (11); the results of correlation analysis of all items were statistically significant, and the degree of correlation was high. In particular, the correlation coefficients of the ‘food approach’ factors FR-EF and FR-EOE were 0.65 and 0.64, respectively, showing strong positive correlations. These results are similar to those of studies examining correlations between factors of the CEBQ for Dutch, Spanish, and Portuguese children regardless of the disease or symptom (12, 13, 21). Accordingly, it is expected that the factors may show a positive correlation regardless of specific diseases and symptoms. Particularly, the EUE factor belonging to the ‘food avoidant’ category showed positive r values of 0.06–0.17 with the ‘food approach’ factors. Similar results have been obtained in studies conducted on children in other countries (12, 13, 21).

Differences in eating behavior according to sex assessed by the K-CEBQ were minimal, except for the SE factor. This finding is consistent with that of a study involving Korean children with normal development (11), suggesting that the eating behavior of children with anorexia develops in a similar manner regardless of sex. On the other hand, the ‘food avoidant’ category score and EUE factor score were significantly higher for children under 6 years of age, thus showing differences according to age. This result is consistent with that of previous studies showing that children’s food refusal problems decreased with age (2, 22). In addition, a recent cohort study revealed that children’s food avoidant traits were negatively associated with age (23). To minimize the effect of this, we performed subgroup analysis according to sex and age when calculating optimal cut-off scores, and the results after PSM according to sex and age are presented separately.

There were significant differences between the anorexia and normal groups in the ‘food approach’ and ‘food avoidant’ before and after PSM, confirming the discriminant validity of the K-CEBQ for the diagnosis of anorexia. Analysis based on individual factors also revealed significant differences between the two groups, except for the EOE, DD, and EUE factors. These results are similar to those of a previous study involving 106 participants, in which there were significant differences except for EOE and DD when comparing children with anorexia and without anorexia (24).

In our study, anorexic children were defined and analyzed by referring to Diagnostic Classification of Mental Health and Developmental Disorders of Infancy and Early Childhood (DC:0–3R); however, the criteria originally applied to children up to 3 years of age (16). Therefore, considering the inclusion of anorexic patients of various ages and severities, cut-off scores for optimally diagnosing anorexia were calculated. In the ‘food avoidant’ category, the optimal cut-off score of the K-CEBQ calculated in our study did not show significant differences in subgroup analysis according to age and sex, indicating the robustness of the results. Therefore, the cut-off score for the ‘food avoidant’ category derived from this study may be used as an auxiliary tool for clinicians to evaluate the progress of anorexia treatment in clinical settings and research. Several studies have been conducted to analyze the association between the CEBQ and BMI (12, 13, 25, 26), demonstrating that the CEBQ is valuable for identifying specific eating styles associated with the weight status of children. Therefore, for the evaluation and management of growing children, the CEBQ can be introduced along with BMI and body composition tests in the primary medical environment, and the cut-off score for the ‘food avoidant’ category may be used as a tool for managing the growth and development of children. However, the cut-off score for the ‘food approach’ category showed differences in subgroup analysis after PSM. In this category, the specificity in diagnostic accuracy was relatively low, and the AUC was between 0.5 and 0.7. This variation might be attributed to the relatively small number of participants in the normal group compared with the anorexia group. Therefore, to obtain more definitive results, the cut-off score for the ‘food approach’ category should be confirmed using a larger sample size in future studies.

There are several limitations in this study. First, this study did not consider various confounding factors (other than sex and age) that may influence the anorexia symptoms of children during group comparisons. Further research considering additional confounding factors would be necessary in the future. Second, the sample size of the normal group was relatively small compared with that of the anorexia group. Therefore, we used sex and age-matched groups to evaluate the results of discriminant validity analysis, including statistical significance and trend, which were not significantly different from those before matching. Finally, as this study was based on an online survey, there may be non-coverage errors and self-selection errors (27).

Despite its limitations, this study is the first to evaluate the reliability and construct validity of the K-CEBQ for children with anorexia. In addition, cut-off scores of the K-CEBQ were calculated for the first time. As the K-CEBQ comprehensively measures not only refusal to eat but also overeating behavior, we attempted to define practical cut-off scores by classifying the factors into the two categories ‘food avoidant’ and ‘food approach’ according to the measurement content. The K-CEBQ may be a useful tool to aid clinical decision-making by evaluating treatment results and progress in clinical settings.

In conclusion, the findings of our study suggest that the K-CEBQ may be a reliable and valid tool for assessing the eating behavior of children with anorexia. The questionnaire showed good internal consistency reliability and temporal stability and was able to differentiate between groups with and without anorexia. Furthermore, the questionnaire was able to measure distinct dimensions of eating behavior for children with anorexia. These results are meaningful as they demonstrated the potential of the K-CEBQ as a reliable and valid evaluation tool that may be used to aid clinical decision-making to diagnose and treat anorexia in children.

## Data availability statement

The raw data supporting the conclusions of this article will be made available by the authors, without undue reservation.

## Ethics statement

The studies involving humans were approved by the Institutional Review Board of the Korea Institute of Oriental Medicine (no. I-2109/008–002, October 13, 2021). The studies were conducted in accordance with the local legislation and institutional requirements. The ethics committee/institutional review board waived the requirement of written informed consent for participation from the participants or the participants’ legal guardians/next of kin because as the study did not collect any identifiable information from the participants.

## Author contributions

SL and BL: conceptualization. MK and BL: methodology. MK: formal analysis. MK and BL: writing – original draft. MK, SL, GC, and BL: writing – review & editing. GC: funding acquisition. All authors contributed to the article and approved the submitted version.

## Footnotes

^1^https://public.embrain.com/, Seoul, South Korea.
