# Supplementary material for: Reliability and validity of a Korean version of the children’s eating behavior questionnaire in anorexia context
Source: Front Nutr. 2023 Nov 1;10:1247630. doi: 10.3389/fnut.2023.1247630 (PMC10646492; doi:10.3389/fnut.2023.1247630)
Supplement: Supplementary file 1 [file Data_Sheet_1.docx]

**Supplements**

**Supplemental Table 1. K-CEBQ scores according to sex**

| **K-CEBQ** | **Total**  **(N = 366)** | **Boys**  **(N = 213)** | **Girls**  **(N = 153)** | ***p*-value** |
| --- | --- | --- | --- | --- |
| ***‘Food approach’*** | 30.98 ± 7.86 | 30.90 ± 8.30 | 31.1 ± 7.22 | 0.8032 |
| Food responsiveness (FR) | 7.84 ± 2.68 | 7.92 ± 2.76 | 7.74 ± 2.56 | 0.5228 |
| Enjoyment of food (EF) | 9.55 ± 2.74 | 9.49 ± 2.85 | 9.63 ± 2.60 | 0.6282 |
| Emotional overeating (EOE) | 5.66 ± 2.2 | 5.64 ± 2.31 | 5.69 ± 2.03 | 0.8534 |
| Desire to drink (DD) | 7.93 ± 3.26 | 7.84 ± 3.29 | 8.05 ± 3.22 | 0.5528 |
| ***‘Food avoidant’*** | 64.09 ± 9 | 63.54 ± 8.69 | 64.86 ± 9.40 | 0.1696 |
| Satiety responsiveness (SR) | 16.92 ± 3.44 | 16.63 ± 3.30 | 17.31 ± 3.06 | 0.0646 |
| Slowness in eating (SE) | 15.77 ± 3.45 | 15.46 ± 3.61 | 16.22 ± 3.18 | **0.0375** |
| Emotional undereating (EUE) | 11.67 ± 3.52 | 11.5 ± 3.39 | 11.92 ± 3.69 | 0.2693 |
| Food fussiness (FF) | 19.73 ± 3.36 | 19.95 ± 3.30 | 19.42 ± 3.43 | 0.1335 |

All data are presented as Mean ± SD. *p*-value by independent t-test. K-CEBQ, the Korean version of Children's Eating Behavior Questionnaire.

**Supplemental Table 2. K-CEBQ scores according to age**

| **K-CEBQ** | **Total**  **(N = 366)** | **< 6 years**  **(N = 184)** | **≥ 6 years**  **(N = 182)** | ***p*-value** |
| --- | --- | --- | --- | --- |
| ***‘Food approach’*** | 30.98 ± 7.86 | 31.78 ± 7.86 | 30.18 ± 7.79 | 0.0519 |
| Food responsiveness (FR) | 7.84 ± 2.68 | 8.02 ± 2.76 | 7.66 ± 2.59 | 0.2027 |
| Enjoyment of food (EF) | 9.55 ± 2.74 | 9.68 ± 2.68 | 9.42 ± 2.81 | 0.3522 |
| Emotional overeating (EOE) | 5.66 ± 2.20 | 5.66 ± 2.25 | 5.66 ± 2.15 | 0.9872 |
| Desire to drink (DD) | 7.93 ± 3.26 | 8.41 ± 3.28 | 7.44 ± 3.18 | **0.0044** |
| ***‘Food avoidant’*** | 64.09 ± 9.0 | 65.12 ± 8.99 | 63.05 ± 8.92 | **0.0281** |
| Satiety responsiveness (SR) | 16.92 ± 3.44 | 16.93 ± 3.46 | 16.90 ± 3.43 | 0.9375 |
| Slowness in eating (SE) | 15.77 ± 3.45 | 15.89 ± 3.37 | 15.65 ± 3.54 | 0.5113 |
| Emotional undereating (EUE) | 11.67 ± 3.52 | 12.27 ± 3.36 | 11.07 ± 3.58 | **0.0010** |
| Food fussiness (FF) | 19.73 ± 3.36 | 20.03 ± 3.15 | 19.43 ± 3.55 | 0.0885 |

All data are presented as Mean ± SD. *p*-value by independent t-test. K-CEBQ, the Korean version of Children's Eating Behavior Questionnaire.

**Supplemental Table 3. Comparison of the K-CEBQ between groups (after age-sex matching)**

| **Variables** | **Total**  **(N = 86)** | **Anorexia**  **(N = 43)** | **Normal**  **(N = 43)** | ***p*-value** |
| --- | --- | --- | --- | --- |
| Sex (M/F) | 42/44 | 21/22 | 21/22 | <.9999 |
| Age (years) | 6.07 ± 1.75 | 6.07 ± 1.82 | 6.07 ± 1.71 | <.9999 |
| **K-CEBQ** |  |  |  |  |
| ***‘Food approach’*** | 31.49 ± 9.06 | 28.53 ± 6.87 | 34.44 ± 10.06 | **0.0021** |
| Food responsiveness (FR) | 8.21 ± 2.94 | 7.28 ± 2.23 | 9.14 ± 3.27 | **0.0029** |
| Enjoyment of food (EF) | 10.24 ± 3.16 | 8.86 ± 2.1 | 11.63 ± 3.45 | **<.0001** |
| Emotional overeating (EOE) | 5.59 ± 2.29 | 4.95 ± 1.62 | 6.23 ± 2.68 | **0.0089** |
| Desire to drink (DD) | 7.44 ± 3.22 | 7.44 ± 3.61 | 7.44 ± 2.81 | <.9999 |
| ***‘Food avoidant’*** | 60.23 ± 9.34 | 63.86 ± 8.73 | 56.6 ± 8.56 | **0.0002** |
| Satiety responsiveness (SR) | 15.78 ± 3.53 | 17.56 ± 3.29 | 14.0 ± 2.81 | **<.0001** |
| Slowness in eating (SE) | 15.34 ± 3.13 | 16.37 ± 3.09 | 14.3 ± 2.84 | **0.0018** |
| Emotional undereating (EUE) | 10.92 ± 3.6 | 10.72 ± 4.02 | 11.12 ± 3.16 | 0.6136 |
| Food fussiness (FF) | 18.2 ± 3.9 | 19.21 ± 3.7 | 17.19 ± 3.88 | **0.0154** |

*p*-value by independent t-test. K-CEBQ, the Korean version of Children's Eating Behavior Questionnaire;

**Supplemental Figure 1. Receiver operating characteristic curve of the K-CEBQ (after age-sex matching)**


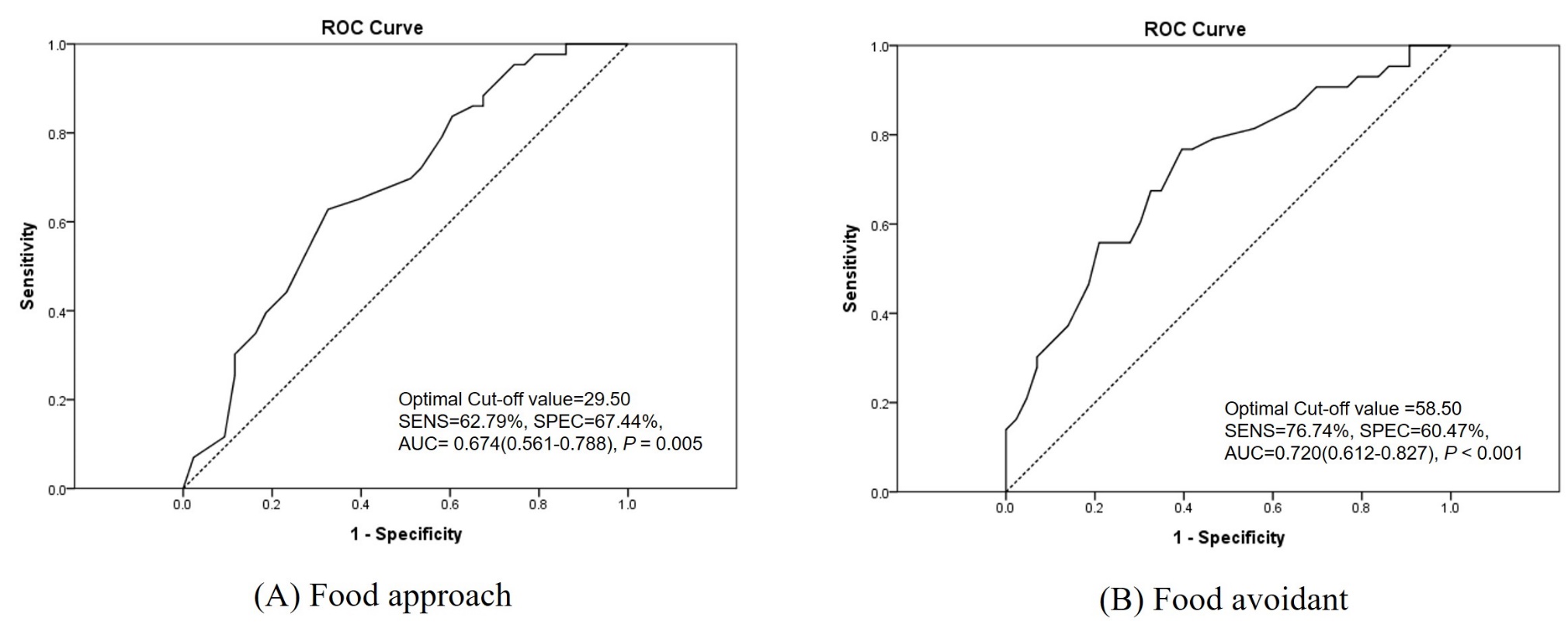


**Supplemental Figure 2. Receiver operating characteristic curve of the K-CEBQ according to sex (after age-sex matching)**

**
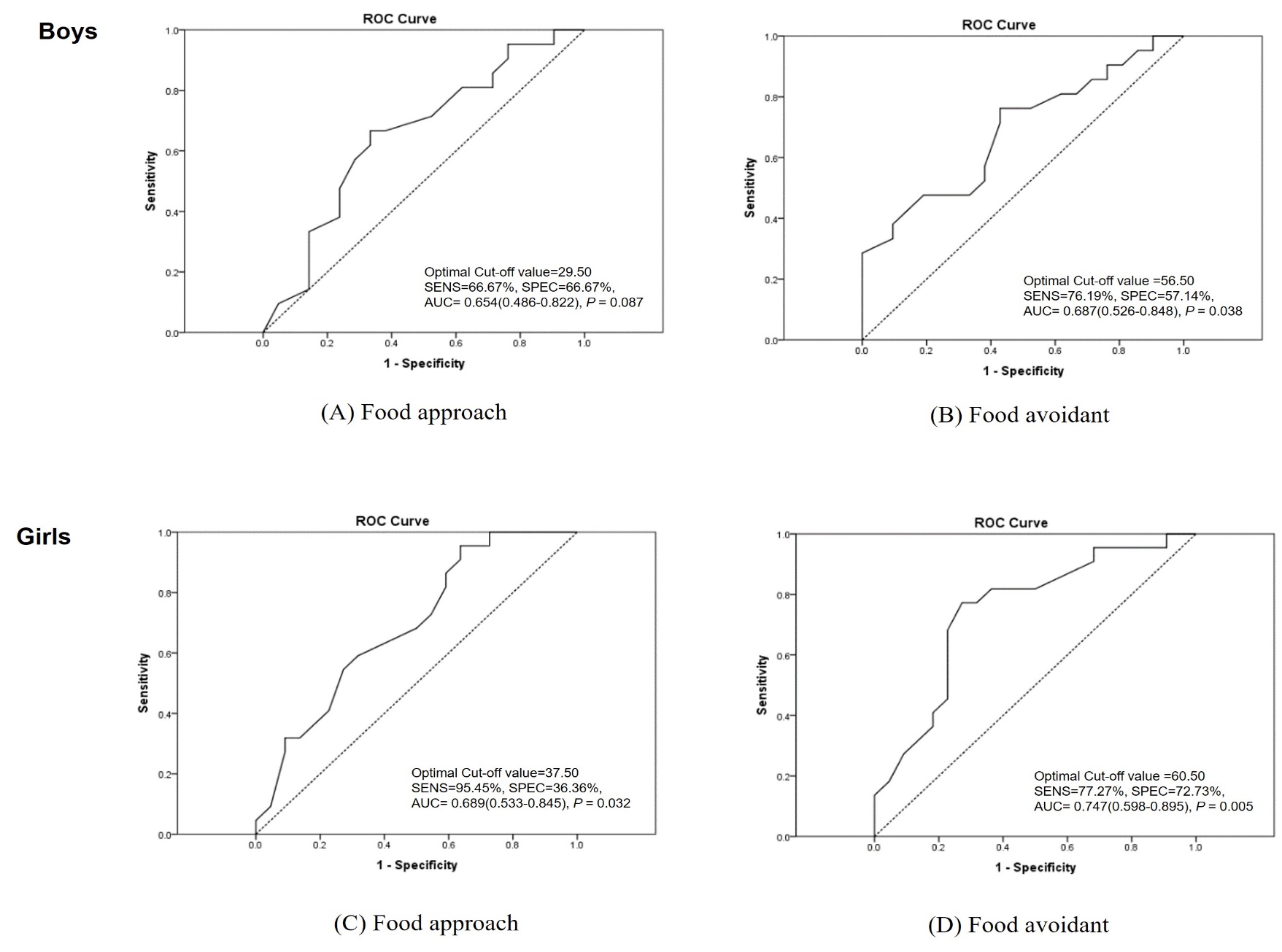
**

**Supplemental Figure 3. Receiver operating characteristic curve of the K-CEBQ according to age (after age-sex matching)**

**
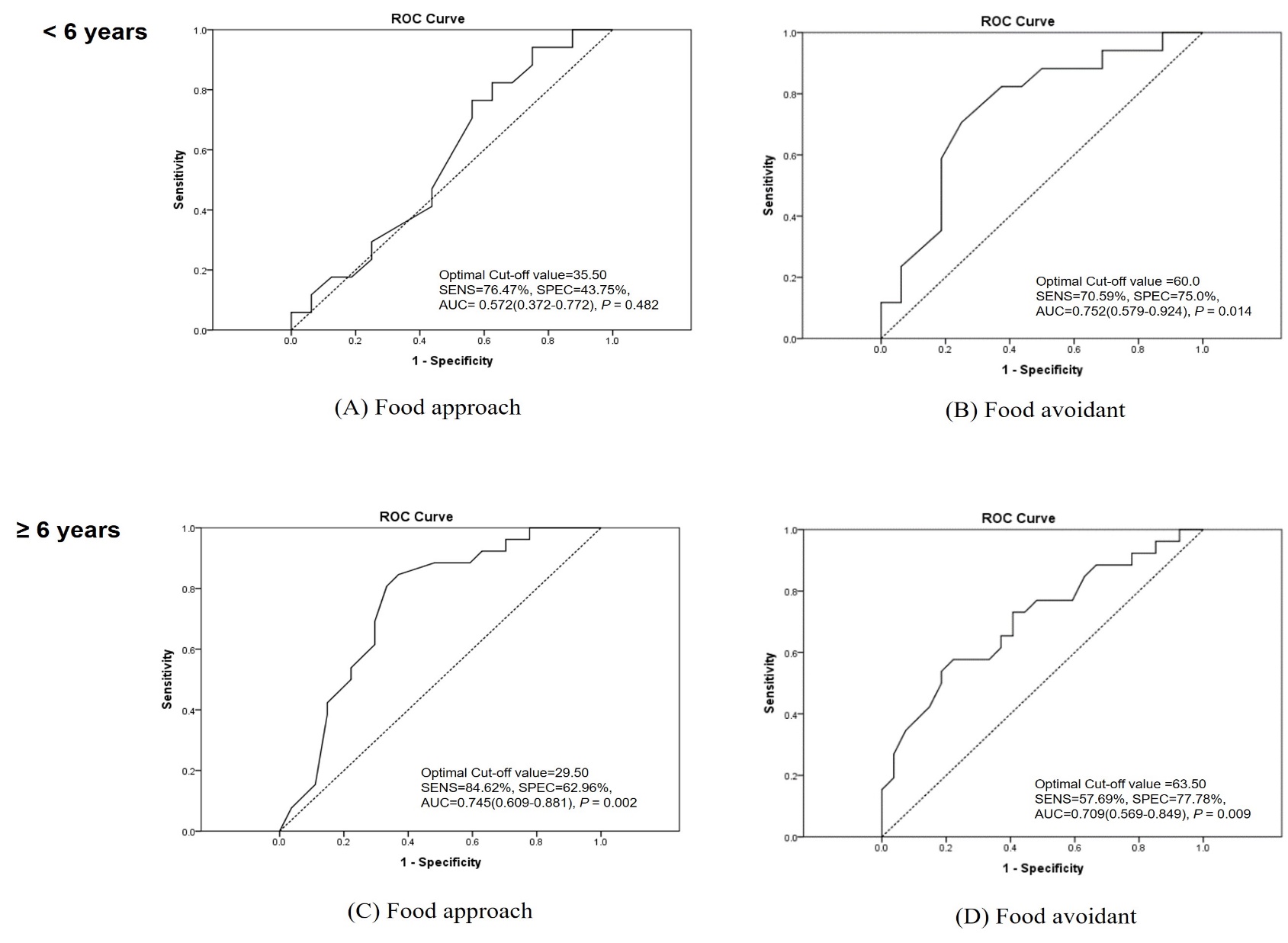
**
